# Niche divergence promotes rapid diversification of East African sky island white-eyes (Aves: Zosteropidae)

**DOI:** 10.1111/mec.12840

**Published:** 2014-07-08

**Authors:** Siobhan C Cox, Robert P Prys-Jones, Jan C Habel, Bernard A Amakobe, Julia J Day

**Affiliations:** * Department of Genetics, Evolution and Environment, University College LondonGower Street, London, WC1E 6BT, UK; † Bird Group, Department of Life Sciences, The Natural History MuseumAkeman Street, Tring, Hertfordshire, HP23 6AP, UK; ‡ Terrestrial Ecology Research Group, Department of Ecology and Ecosystem Management, Technische Universität München, Hans-Carl-von-Carlowitz-Platz 2D-85354, Freising-Weihenstephan, Germany; § Ornithology Section, Department of Zoology, National Museums of KenyaP.O. Box 78420-00500, Nairobi, Kenya

**Keywords:** AFLPs, Afromontane, biodiversity hotspot, molecular dating, montane diversification, Plio-Pleistocene

## Abstract

The Eastern Afromontane biodiversity hotspot composed of highly fragmented forested highlands (sky islands) harbours exceptional diversity and endemicity, particularly within birds. To explain their elevated diversity within this region, models founded on niche conservatism have been offered, although detailed phylogeographic studies are limited to a few avian lineages. Here, we focus on the recent songbird genus *Zosterops,* represented by montane and lowland members, to test the roles of niche conservatism versus niche divergence in the diversification and colonization of East Africa's sky islands. The species-rich white-eyes are a typically homogeneous family with an exceptional colonizing ability, but in contrast to their diversity on oceanic islands, continental diversity is considered depauperate and has been largely neglected. Molecular phylogenetic analysis of ∼140 taxa reveals extensive polyphyly among different montane populations of *Z. poliogastrus*. These larger endemic birds are shown to be more closely related to taxa with divergent habitat types, altitudinal distributions and dispersal abilities than they are to populations of restricted endemics that occur in neighbouring montane forest fragments. This repeated transition between lowland and highland habitats over time demonstrate that diversification of the focal group is explained by niche divergence. Our results also highlight an underestimation of diversity compared to morphological studies that has implications for their taxonomy and conservation. Molecular dating suggests that the spatially extensive African radiation arose exceptionally rapidly (1–2.5 Ma) during the fluctuating Plio-Pleistocene climate, which may have provided the primary driver for lineage diversification.

## Introduction

Fragmented landscapes such as archipelagos are excellent natural laboratories to assess the influence of geography on genetic and phenotypic divergence (e.g. Roy 1997; Knowles 2000; McCormack *et al*. 2008; Price 2008; Shepard & Burbrink 2009; Clegg & Phillimore 2010; Lawson 2013). In particular, montane archipelagos harbour some of the highest biological diversity on the planet, making them important regions of interest for understanding the patterns and processes leading to the accumulation of diversity. The hyperdiverse Eastern Afromontane biodiversity hotspot (EABH) (Myers *et al*. 2000) has received considerable attention from evolutionary biologists investigating how this highly heterogeneous landscape has influenced population differentiation and speciation both temporally and spatially. This region is currently experiencing severe habitat loss (Myers *et al*. 2000) and has alarming rates of forecasted urban growth (Seto *et al*. 2012), which places a premium on quantifying the diversity that this key hotspot harbours, as well as understanding the evolutionary processes responsible. Evolutionary insights will furthermore allow a better understanding of organisms' responses to on-going and future habitat fragmentation.

**Figure 1:**
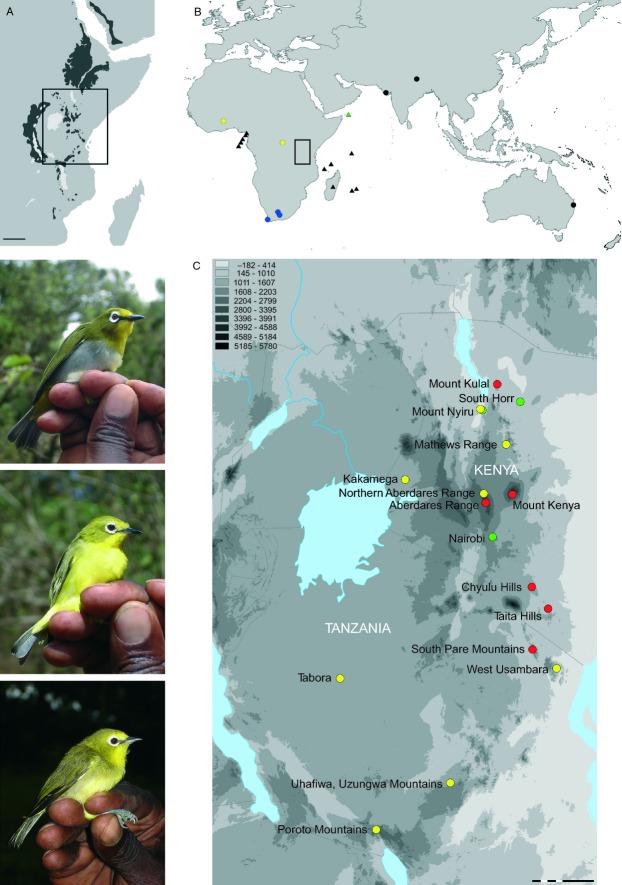
Distribution of Zosteropidae samples used in this study. (A) The Eastern Afromontane region (highlighted in black), scale = 500 Km; (B) *Zosterops* samples from outside the focal region; (C) *Zosterops* samples from the East Afromontane region: *Z. poligastrus* (red); *Z. abyssinicus* (green); *Z. senegalensis* (yellow); *Z. pallidus* (blue); *Zosterops* sp. (black); mainland taxa (circles); insular taxa (triangles); altitude in metres; scale=3.2 decimal degrees. Photographs (top to bottom, Cox): *Z. poliogastrus kulalensis* (K39, Mt Kulal); *Z. abyssinicus flavilateralis* (T15, foothill of Chyulu Hills); *Z. senegalensis jacksoni* (T51, Kakamega Forest).

Unlike the continuous mountain ranges of the Himalayas or Andes, the EABH is composed of a chain of ancient isolated massifs (Griffiths 1993) and young volcanoes (<5 Ma e.g. Baker *et al*. 1971) forming sky islands (Fig.1A). Montane forests typically occur above 800 m on these isolated peaks, so that the climatic conditions and ecosystem are highly differentiated from the surrounding low altitude savannah habitats and thus form ‘ecological islands’. Previously, the montane forests formed a pan-African forest that fragmented in the Early Oligocene due to the onset of aridification (Lovett 1993; Sepulchre *et al*. 2006) and have therefore had a long period of isolation. The isolation of these habitats potentially allows in situ speciation events to be differentiated from colonization events, which would otherwise be much harder to identify in montane systems exhibiting higher degrees of connectivity (Voelker *et al*. 2010).

Several competing models have been put forward in an attempt to explain the high levels of endemism observed in tropical montane faunas. Within the EABH, there has been considerable support for the montane speciation model (e.g. Fjeldså & Lovett 1997; Roy 1997; Fjeldså & Bowie 2008). Under this scenario, montane forest habitats (sky islands) separated by intervening lowland areas may have served as historical refugia, where previously widespread populations became geographically isolated as they tracked suitable habitat to higher altitudes in response to climate change during the cool and arid episodes of the Plio-Pleistocene.

An alternative mechanism of climatic zonation, the gradient speciation model (Moritz *et al*. 2000 and references therein), posits that new species originate as populations adapt to different climatic regimes along an altitudinal gradient, predicting that sister taxa should occupy distinct but adjacent habitats (e.g. Moritz *et al*. 2000; Ogden & Thorpe 2002; Hall 2005; Kozak & Wiens 2007). In the tropics, the narrowing of climatic profiles between different altitudes produces strong ecological gradients, which in turn selects organisms with narrow ecological tolerances (Moritz *et al*. 2000; Kozak & Wiens 2007).

The montane and gradient speciation models predict contrasting roles for natural selection, with refuge (i.e. montane speciation) models founded on niche conservatism, in which the inability of populations to adapt to new or changing environmental conditions plays the primary role in geographical isolation, with ecologically similar populations diverging in allopatry (Moritz *et al*. 2000; Wiens & Donoghue 2004; Kozak & Wiens 2007; Wiens *et al*. 2010). In contrast, under the gradient model, the ability to adapt to new or changing environmental conditions drives climatic niche divergence (thus population divergence), with differing climatic distributions and/or climatic tolerances limiting gene flow between populations in either allopatry or parapatry (Moritz *et al*. 2000; Ogden & Thorpe 2002; Hall 2005; Kozak & Wiens 2007). A variation of these two models, the vanishing refuge model (Vanzolini & Williams 1981), proposes that some populations speciate through directional selection towards a tolerance of less favourable habitats as refuges become too small to retain viable populations. Like the gradient model, the vanishing refuge model is based on niche divergence and predicts that sister species occupy distinct habitats; however, the latter model also requires severe population bottlenecks with subsequent range expansion (Moritz *et al*. 2000). However, the possible contribution of models founded on niche divergence to explain the diversification of lineages from the EABH has been largely ignored.

Birds have long been the subjects of speciation studies on archipelagos due to their success as colonizers (e.g. Mayr 1942; Diamond 1970; Price 2008; Clegg & Phillimore 2010), with studies principally focusing on oceanic islands and to a lesser extent on montane archipelagos (but see e.g. McCormack *et al*. 2008). Within the EABH, there are approximately 1300 described bird species, of which 110 are endemic, providing substantial comparative systems in which to investigate processes facilitating diversification across the region's sky islands.

To better understand the build-up of biodiversity within the Eastern Afromontane region, we investigated diversification in the songbird genus *Zosterops* (Passeriformes: Zosteropidae). This species-rich group is composed of small, gregarious, arboreal birds that, aside from some aberrant African taxa traditionally separated in the genus *Speirops* (e.g. Melo *et al*. 2011), exhibit remarkable uniformity in their morphological structure, plumage and behaviour (van Balen 2008). As such, the systematics of the Zosteropidae is notoriously problematic, and the affinities of numerous taxa remain enigmatic (Fry *et al*. 2000). Here, we follow the currently standard taxonomy of Dickinson (2003) and van Balen (2008).

*Zosterops* are renowned ‘speciators’, displaying spectacular colonizing abilities (Lack 1971), and are suggested to have diversified exceptionally rapidly (∼2 Ma, Moyle *et al*. 2009). Despite their occurrence across the Old World tropics (including Africa and Asia), research into factors facilitating their diversification has predominately centred on insular taxa (e.g. Warren *et al*. 2006; Moyle *et al*. 2009; Clegg & Phillimore 2010). Conversely, continental members have been largely neglected, possibly because less than 10% of their known diversity is attributed to continental landmasses (Moreau 1957).

Where present on East African sky islands (Fig.1), *Zosterops* (referred to as white-eyes due to their white-eye ring) are often represented by a single endemic subspecies, which is analogous to their distribution on oceanic islands (e.g. Indian Ocean, Gulf of Guinea and Vanuatu archipelagos). Oceanic islands commonly support a single endemic taxon (Dickinson 2003), although two nonsister taxa are present on some islands (e.g. Warren *et al*. 2006). Just four currently recognized species of *Zosterops* occur across much of mainland sub-Saharan Africa, of which three are found within the mountain archipelago of East Africa's Rift Valley (Kenyan Highlands and East African Arc); however, subspecies diversity is considerably higher (Dickinson 2003; van Balen 2008). The African montane white-eyes (*Z. poliogastrus*) of East Africa's sky islands are comparatively larger birds than other mainland species, with rich green backs, yellow or grey bellies and generally broad white-eye rings and bright golden feathers (Fig.1). While some authors have argued that *Z. poliogastrus* should be split into several species based on vocal differences and ecology (e.g. Collar *et al*. 1994; Borghesio & Laiolo 2004), plumage variation within this group is subtle. Notably, a recent study based on 15 microsatellite markers indicates that genetic differentiation within *Z. poliogaster* populations is very high (Habel *et al*. 2013). All *Z. poliogastrus* subspecies are endemic to montane forest habitats and are ecologically segregated from parapatric *Z. senegalensis* or *Z. abyssinicus* subspecies (Hall & Moreau 1970).

The recent divergence of the Zosteropidae (Moyle *et al*. 2009) and their montane and lowland distribution within the focal region are attributes that make East African *Zosterops* a useful system to test current hypotheses of montane diversification and diversity using a phylogenetic approach. An advantage of focusing on recently evolved taxa is that they avoid complicating causal events; thus, their diversity is likely shaped by similar forces happening in a short amount of time, rather than multiple disparate events over a long time. To reconstruct the evolutionary relationships of *Zosterops*, we generated novel mtDNA (cytochrome *b* (Cyt *b*) and NADH dehydrogenase subunit 3 (ND3) genes), and AFLP data based on dense sampling across the Eastern Afromontane region and included additional sampling from outside of the region. Divergence times were estimated using a calibration based on the age of a volcanic island (Grand Comore) and were compared to those generated using the avian molecular clock. Using these data, we address the following questions: First, Have montane forest taxa diversified in situ? Under this scenario, diversification of montane endemics is suggested to be the result of niche conservatism (montane speciation model), in which we would except to find recently evolved montane forms (Plio-Pleistocene time frame) comprising a clade relative to lowland forms (e.g. Roy 1997). In contrast, alternative hypotheses in which diversification is the result of niche divergence (gradient speciation and vanishing refuge models), we would expect sister species to occupy distinct habitats. However, as the latter model requires demographic changes to explain such shifts, we are unable to test this hypothesis here. Second, Did diversification of montane taxa occur within a Plio-Pleistocene time frame, in which montane habitats have been largely buffered from climatic instability? Third, Is montane white-eye diversity underestimated? That a species-rich genus contains so few species in a key hotspot of biological diversity is surprising. Extensive sampling of the region here allows assessment of their diversity within a systematic framework.

## Methods

### Taxonomic sampling

A total of 148 individuals representing 15 described ingroup taxa (Dickinson 2003) are included in this study based on blood samples (Table S1, Supporting information; Fig.1). To test species monophyly and colonization scenarios, 33 *Zosterops* samples were obtained from outside East Africa (Fig.1B). Blood sample numbers, collection localities and GenBank Accession nos are listed in Table S1 (accompanying specimen photographs are available from Dryad). Blood samples were taken from mist-netted specimens and stored in ETOH (99%) or Queen's lysis buffer (Seutin *et al*. 1991).

### Molecular sequence data

Total DNA was extracted from blood samples using a DNeasy Blood and tissue kit (Qiagen, UK). Mitochondrial genes (Cyt *b*, ND3) and a nuclear gene (TGFß2) were selected based on their performance from previous *Zosterops* studies (Warren *et al*. 2006; Moyle *et al*. 2009; Melo *et al*. 2011). Amplification of ND3 and TGFß2 was performed using published primers (Table S2, Supporting information). To obtain the entire Cyt *b* gene, the published primer H16065 was used alongside three newly designed primers (Table S2, Supporting information). PCR amplifications and thermal cycling conditions for all three genes are reported in Table S2. Cleaned products were sequenced on an ABI 3730xl DNA analyser (Applied Biosystems, UK).

### Amplified Fragment Length Polymorphisms (AFLPs)

Amplified fragment length polymorphism profiles were generated following Vos *et al*. (1995), with modifications for fluorescent primers implemented in Huang & Sun (1999). Selective PCR was carried out in two stages: a subsample of all restriction fragments was obtained through a preselective amplification, and these were subsequently selectively amplified with more specific dye labelled primers. Thermal cycling conditions and primers for both amplification stages are reported in Appendix S1, Supporting information. This study screened a total of 21 unique primer combinations generated from three selective amplification EcoRI+NNN primers (labelled with different fluorescent dyes) and seven Msel+NNN primers. A subset of eight DNA extracts was chosen to test all 21 AFLP primers combinations, and resulting selective amplification products were electrophoresed on a 3.5% agarose gel against a Hyperladder V (Bioline) size standard to choose the most appropriate primer combinations. Fragment analysis was conducted on a 3730 Applied Biosystems Sanger Sequencer using recommended fluorophores (FAM, NED HEX and LIZ).

### AFLP scoring

Peaks were visualized using genemapper version 3.7, and all primer combinations were analysed separately. An initial scoring panel was generated using the automatic panel generation feature of genemapper under default settings. This feature algorithmically generates panels and bins based on the collective peaks present from all samples. The resulting AFLP panels were then checked by eye. As replicates are the only objective measure of quality in AFLP studies (Pompanon *et al*. 2005), five individuals were repeated from the restriction ligation stage onwards to obtain a relative assessment of the repeatability of AFLP profiles. Additional information is reported in Appendix S1.

### Phylogenetic inference

Phylogenetic analyses were performed on a data set of 139 samples comprising 1471 bp of mtDNA sequence data (ND3 348 bp, Cyt *b* 1123 bp). Sequences were aligned in clustal x 2.0 (Larkin *et al*. 2007) using default settings, with the resulting alignment checked manually in se-al 2.0 (Rambaut 2002) and translated into amino acids to ensure there were no stop codons. Nuclear DNA (TGFß2) (600 bp) was generated for a subset of the taxa to assess phylogenetic signal, but provided no informative sites and was therefore discounted from subsequent analyses.

partitionfinder 1.01 (Lanfear *et al*. 2012) was used to select the best-fit partitioning scheme and model of molecular evolution for the mtDNA data using the Bayesian information criterion (BIC) and implementing a heuristic search algorithm (greedy). The resulting partitions and models were implemented in mrbayes 3.1.2 (Huelsenbeck & Ronquist 2001). Starting from a random tree, four metropolis-coupled Markov chain Monte Carlo (MCMC) chains (temp = 0.2) were run simultaneously for 5 000 000 generations three times, sampling every 100 generations with a burn-in of 7500. Convergence of the MCMC runs was assessed graphically using tracer 1.5 (Rambaut & Drummond 2009). Support is assessed by Bayesian posterior probabilities (BPP). Maximum-likelihood (ML) analyses were also performed on the mtDNA data and implemented in garli (Genetic Algorithm for Rapid Likelihood Inference 2.0) (Zwickl 2006). Six search replicates were run to find the best tree, estimating substitution rates, with branch support ascertained by 1000 nonparametric bootstrap (BS) replicates.

A phylogenetic analysis of the 255 character AFLP data based on 92 samples was also performed using mrbayes 3.1.2. Four independent MCMC chains (temp = 0.2) were run for 5 000 000 generations, with a sampling frequency of 1000 and a relative burn-in of 25%. The binary matrix was coded as data-type=restriction and coding=no absence sites, with all other parameters set as default, with support estimated by BPP.

### Estimation of divergence times

As there are no suitable fossil calibrations for the Zosteropidae, we employed alternative methods of molecular dating. This included using a geological calibration based on the date of origin of a volcanic island, an approach that has been employed in various other avian studies (e.g. Fleischer *et al*. 1998; Warren *et al*. 2006; Moyle *et al*. 2009; Lerner *et al*. 2011; Melo *et al*. 2011). Under this approach, the maximum age of divergence between closely related taxa occupying neighbouring islands is constrained to be the age of the youngest island, representing the earliest possible date for colonization. Following assumptions discussed in previous studies (e.g. Fleischer *et al*. 1998; Warren *et al*. 2003), the maximum age estimate for the volcanic origin of Grande Comore at 0.5 Ma (R. Duncan personal communication in Warren *et al*. 2003) is used to calibrate the node separating the lowland Grande Comore white-eye (*Z. maderaspatanus kirki*) from other taxa in the ‘*maderaspatanus*’ clade (Warren *et al*. 2006). As an avian molecular clock has been used to date many avian studies (e.g. Voelker *et al*. 2010; Fritz *et al*. 2011), we also employed the average pairwise substitution rate of 2.1% for Cyt *b* (Weir & Schluter 2008) used in these studies to investigate whether resulting divergence times are concurrent with those generated from an island calibration.

Cytochrome *b* and ND3 data were pruned to include one representative from each taxon in order to use a model of speciation with no coalescence and were found to be evolving under a strict molecular clock in beast 1.7.5 (Drummond *et al*. 2012). In the calibrated approach, divergence time estimates were generated from the concatenated Cyt *b* and ND3 data, while divergence estimates obtained based on the molecular clock rate (2.1%) were generated from the Cyt *b* data only. Both approaches used the same starting tree that was generated from the Bayesian analyses of the concatenated data, but retaining only a single sample per taxon. For both analyses, two independent MCMC analyses were run starting from a user specified tree. Chains were run for 10 000 000 generations using a constant rate Yule speciation prior (assumes a constant speciation rate per lineage) implementing the models and partitions generated by partitionfinder, sampling every 1000 generations with a burn-in of 10%. Convergence of the two independent MCMC runs was assessed in tracer 1.5 (Rambaut & Drummond 2009), as were convergence of model parameter values (effective sample size [ESS]) to ensure ESS values >200. The posterior distribution was summarized in the program tree annotator 1.7.5 (Drummond *et al*. 2012). An empty alignment was also run to investigate the effects of priors on posterior divergence and resulted in no priors needing to be updated.

### Biogeographic analysis

To reconstruct whether ancestral areas at nodes for selected clades (A1, A2, B1) in the phylogeny are lowland or highland in origin, we use the event-based method statistical dispersal–vicariance analysis (S-DIVA, Yu *et al*. 2010) in the program Reconstruct Ancestral States in phylogenies v1.1 (RASP; Yu *et al*. 2013) using the pruned tree containing one representative from each taxon.

## Results

### Phylogenetic inference (mtDNA)

Three partitions were identified using PartitionFinder for the concatenated mtDNA with the following models implemented in mrbayes: K80 + I+G (ND3 position 2, Cyt *b* position 3); HYK+I+G (ND3 position 3, Cyt *b* position 1); GTR+G (ND3 position 1, Cyt *b* position 2).

**Figure 2:**
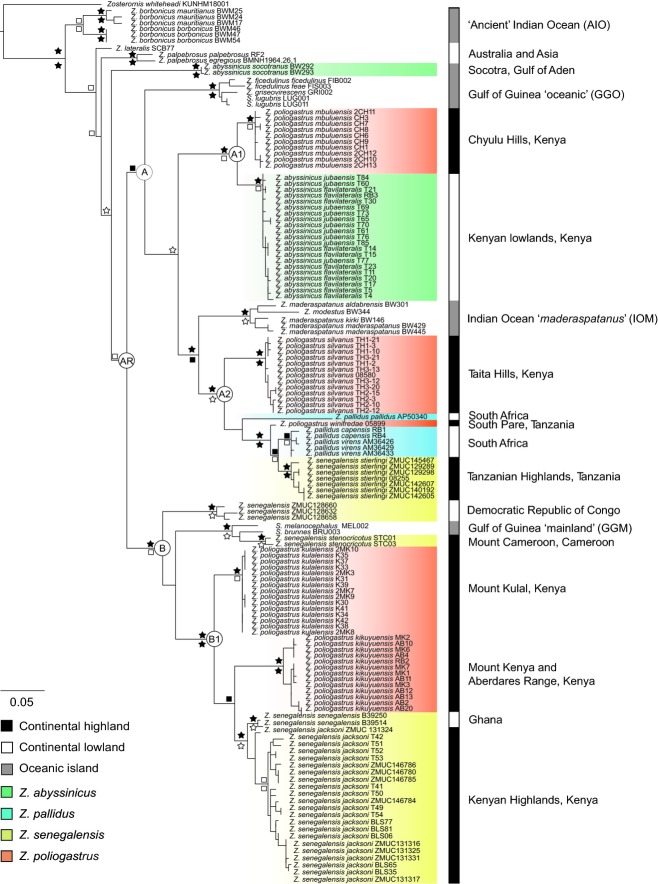
Phylogenetic tree of African *Zosterops* generated using Bayesian inference based on ND3 and Cyt *b* data. Bayesian posterior probabilities (BPP) are indicated above branches, with ML bootstrap (BS) values below branches. Support values are represented by the symbols: black star >95% BPP/BS, white star >90% BPP/BS, black square >80% BPP/BS, white square >50% BPP/BS. Nodes with <50% support are unmarked. Key nodes are labelled AR (African Radiation), A-A2 and B-B1. Taxa are labelled using full trinomial nomenclature following the taxonomy of Dickinson (2003).

Phylogenetic inference based on Bayesian and ML analyses resulted in well-resolved congruent hypotheses in which support for relationships is generally good (Fig.2). Our results identify an African radiation composed of two major clades (Fig.2A, B) that each contains independent oceanic island clades, with the notable exception of the Ancient Indian Ocean white-eyes (AIO) clade that is not a member of this radiation.

A significant and striking finding of this study is the extensive nonmonophyly of mainland African *Zosterops* taxa, with all continental species (*Z. poliogastrus, Z. senegalensis*, *Z. abyssinicus*, *Z. pallidus*) recovered as nonmonophyletic. In contrast to the nonmonophyly of described species, there is strong support for the monophyly of subspecies, specifically within the regional endemic *Z. poliogastrus* and the more widely distributed *Z. senegalensis*.

Each *Z. poliogastrus* and *Z. senegalensis* subspecies sampled in this study comprises a well-supported clade, forming independent lineages throughout both major mainland clades (Fig.2, clades A and B). By contrast, two of the three *Z. pallidus* subspecies (*Z. p. capensis* and *Z. p. virens*) comprise a clade (BPP 0.89, BS 70%) with no internal resolution. The position of the single sample of *Z. p. pallidus* (AP50340) is unclear, although there is no support for its placement as sister to the other Z. *pallidus* subspecies, supporting the findings of a recent phylogeographic study (Oatley *et al*. 2012). Likewise, the two mainland parapatric *Z*.* abyssinicus* subspecies form a clade (BPP 1, BS 75%) with no support for any division between them. However, our results clearly identify that these mainland *Z*.* abyssinicus* taxa are distinct from the insular race *Z. a. socotranus* from the Island of Socotra. Both Bayesian and ML analyses place *Z. a. socotranus* as sister to the African radiation, although this finding does not receive high support.

### Divergence estimates

**Figure 3:**
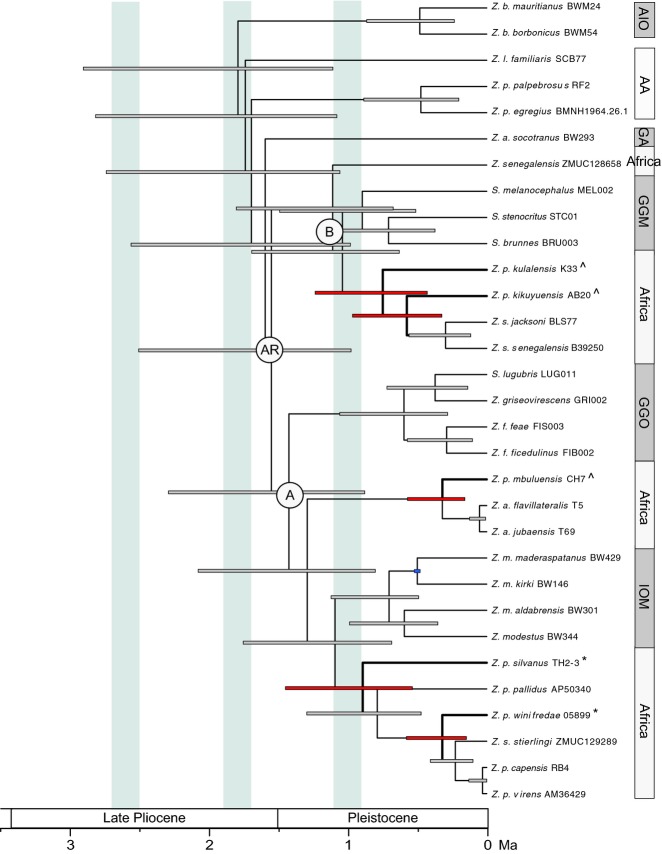
Divergence times of *Zosterops* estimated using beast based on the mitochondrial data and calibrated using a geologically determined island date fixing the node indicated by the blue 95% HPD bar at 0.5 Ma following Warren *et al*. (2006). Thickened branches indicate *Z*. *poliogastrus* taxa, and red HPD bars highlight divergence estimates of the focal taxa. Pale blue bars indicate the warmer and wetter periods of the Plio-Plistocene climate following dates from Trauth *et al*. (2005). AIO, Ancient Indian Ocean; AA, Australia and Asia; GA, Gulf of Aden; GGM, Gulf of Guinea Mainland; GGO, Gulf of Guinea Oceanic; IOM, Indian Ocean ‘*maderaspatanus*’.

The application of different dating methods to the mitochondrial data (volcanic island calibration versus a 2.1% clock rate) results in divergent time frames for the radiation of African *Zosterops*. Implementation of the island calibration (Fig.3) estimates a Late Pleistocene divergence of 1.55 Ma (95% highest posterior density (HPD): 0.97–2.5 Ma). This is considerably younger than the time frame estimated by implementing the 2.1% clock (5.79 Ma 95% HPD: 4.8–6.86 Ma, Fig. S1, Supporting information) pushing the divergence of this clade back to the beginning of the Early Pliocene/Late Miocene. Previous estimates of the molecular rate of evolution in Zosteropidae have used island calibrations (Warren *et al*. 2006; Moyle *et al*. 2009; Melo *et al*. 2011) that have resulted in significantly faster rates of evolution than the 2.1% rate, suggesting that the avian clock may be a severe underestimation of the true rate of evolution within the Zosteropidae. Under the avian clock timescale, the divergence of the clade containing Grande Comore taxa *Z. maderaspatanus kirki* and *Z. maderaspatanus maderaspatanus* is dated at 1.68 Ma (95% HPD: 0.61–2.85 Ma), with a lower confidence interval marginally outside of the island age calibration of 0.5 Ma for the volcanic origin of Grande Comore.

Despite divergence estimates based on median ages for the calibrated tree of the African *Zosterops* radiation falling within a drier climatic phase (Fig.3), we cannot place much confidence in this finding as 95% HPDs are broad at nodes along the backbone of the tree. However, more confidence can be given to the diversification of montane white-eyes during the cooler and drier East African climate (Fig.3) as our timetree indicates two pulses of diversification of these taxa with *Z. poliogastrus silvanus* 0.89 Ma (95% HPD: 0.53–1.45 Ma), *Z. p. kulalensis* 0.75 Ma (95% HPD: 0.43–1.23 Ma) and *Z. p kikuyuensis* 0.57 Ma (95% HPD: 0.32–0.96 Ma) diverging earlier than the contemporaneous divergence of *Z. p. winifredae* and *Z. p mbuluensis* at 0.32 Ma (0.15–0.58 Ma 95% HPD). Diversification of these montane taxa appears to have occurred sometime after the last major wet phase at 1.1–0.9 Ma (Trauth *et al*. 2007), although 95% HPDs for *Z. p. kulalensis* and *Z. p. silvanus* extend across this last wet phase and into the earlier arid phase. Irrespective of dating method, there appears to be little evidence that diversification events correspond to volcanic formation, as these pulses of diversification include members inhabiting both massif (old) and volcanic (young) mountains (Fig.3).

### Biogeographic history

While our phylogeny does not include complete sampling, biogeographic analysis indicates at least one instance in which montane forest habitats are ancestral to lowland habitats (clade B1 100%; clade A1 50% + 50% equal probability that highland+lowland are ancestral), whereas ancestral reconstructions are ambiguous for clade A1 (lowland+highland habitats equally probable). However, at a broader scale, lowland habitats are reconstructed as being ancestral for the African radiation.

### AFLP profiles

In total, 116 samples were screened to determine template DNA quality and quantity, with 27 dropping out due to poor quality extracts and high levels of noise affecting efficient scoring. The number of bins (alleles) for each of the 15 primer combinations identified by the initial scoring panel ranged from 211 to 563. In general, NED-labelled primer combinations gave the fewest number of fragments, while FAM-labelled primer combinations gave the highest. Average peak amplitude was relatively uniform across primer combinations (∼800 RFU), although the range of peak amplitude varied significantly between bins (100–5000). Shoulder stuttering was present in 11 of the 15 primer combinations used and was most frequently observed for FAM-labelled primers. The signal-to-noise ratio was lowest in FAM-labelled primers and was notably higher in HEX- and NED-labelled primers respectively.

Manual examination of concatenated AFLP profiles identified a large variation in peak amplitude between samples, which subsequently led to a large proportion of the bin being discounted (∼90%). Codominant alleles were evident across all primer combinations examined. However, peak amplitude variability between samples hindered assessments of frequency. Average estimates of genotyping error, measured following Bonin *et al*. (2004), were 0.8%. The number of fragments scored per sample ranged from 45 to 84, with the mean number of fragment scored being 66.9. Of the 255 AFLP loci examined, 31% (79 alleles) corresponded to private alleles, for which scoring was limited to individuals from the same sampling locality.

### AFLP phylogenetic inference

**Figure 4:**
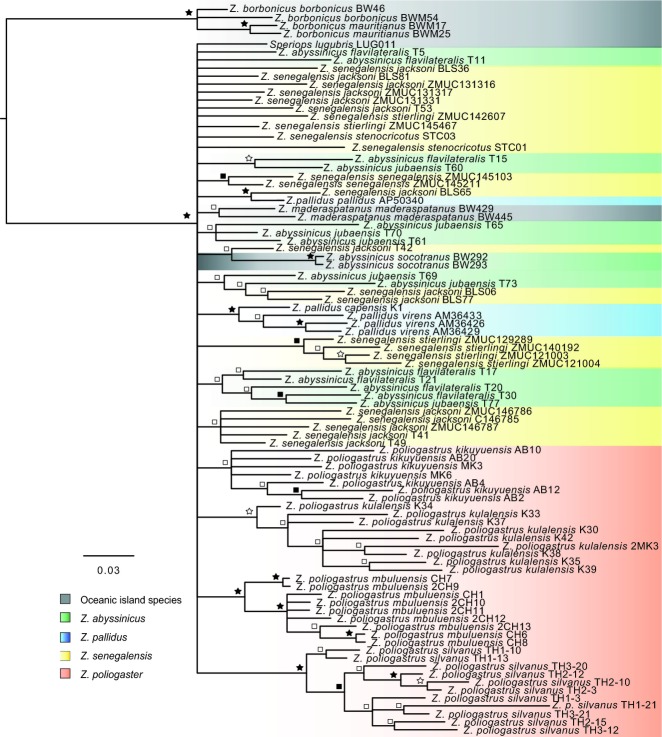
Phylogenetic reconstruction of African *Zosterops* generated by Bayesian inference based on nuclear AFLP fragments. Bayesian posterior probabilities support indicated by the symbols: black star >95%, white star >90%, black square >80% and white square >50%.

Bayesian inference of the AFLP data identified the two *Z. borbonicus* subspecies (Ancient Indian Ocean, AIO) as sister to all Africa taxa (BPP = 1.00, Fig.4), which is concordant with the mtDNA phylogeny. However, there is no support for the broader clades (A and B) recovered in the mtDNA phylogeny. Despite the lack of power in the AFLP data to resolve interrelationships, there is good support for the monophyly of range-restricted taxa of continental montane forests (i.e. *Z. poliogastrus* subspecies). Conversely, there is very little support for the monophyly of subspecies and/or populations of more widely distributed taxa such as *Z. abyssinicus* and *Z. senegalensis*. Leaf stability (Thorley & Wilkinson 1999), implemented in the program Phyutility (Smith & Dunn 2008), was used to assess if the lack of resolution in the initial data (92 taxa) was caused by unstable or rogue taxa. On the basis of leaf stability scores, 14 taxa were discarded, and while the resulting analysis of the reduced matrix resulted in a slightly more resolved hypothesis indicating the nonmonophyly of *Z. poliogastrus,* this result was only weakly supported. Taxon reduction made little impact in resolving deeper level relationships or subspecies monophyly of wide ranging taxa.

## Discussion

### Evidence for niche divergence

We show for the first time using phylogenetic inference that montane white-eyes from East African sky islands are nonmonophyletic. In contrast to other regional studies that have focused on groups containing highland and lowland members at a similar timescale (e.g. Roy 1997; Voje *et al*. 2009), white-eyes have independently colonized but have not subsequently diversified within montane forest habitats. The independent colonization and lack of in situ diversification in an insular setting are generally analogous to white-eyes' history on oceanic islands. The repeated transition that has occurred over evolutionary time between lowlands and highlands strongly indicates a lack of niche conservatism. Additionally, irrespective of absolute dates, our timetree (Fig.3) indicates several instances in which endemic forest taxa are not younger than lowland taxa, which is in contrast to previous findings (e.g. Roy 1997; but see Marks 2010), and that for at least one clade, colonization of East African lowlands is suggested to have occurred by montane species. Thus, although the diversification of the focal group is suggested to have occurred rapidly during the Plio-Pleistocene, the phylogenetic hypothesis does not fit a montane speciation (refuge) model as proposed for other regional lineages (e.g. Roy 1997; Voje *et al*. 2009). Conversely, our results show that the larger and heavier endemic montane *Z. poliogaster* populations are more closely related to taxa with divergent habitat types, altitudinal distributions and dispersal abilities than they are to populations of restricted endemics that occur in neighbouring montane forest fragments. This is exemplified by the endemic montane *Z. p. mubulensis* (Chyulu Hills, Kenya) as sister to a clade comprising *Z. a. flavilateralis* and *Z. a. jubaensis* (Fig.2, clade A1) that have a wide distribution throughout the dry and arid lowlands of Kenya and Ethiopia.

Divergent selection is potentially also further indicated in the phylogeny (clades A2, B1, Fig.2), although relationships are more complex regarding the ecology of sister species. Within each clade, two endemic *Z. poliogastrus* subspecies occur in neighbouring montane forest fragments, with at least one of these subspecies sister to a widely dispersed lowland taxon. Despite the proximity of the forest fragments inhabited by the *Z. poliogastrus* subspecies in clade A2 (<50 km between Taita Hills and S. Pare Mts, and <100 km between Mt Kulal and N. Aberdares), the divergence between these taxa supports the idea that lowland savannah habitat provides a barrier to gene flow causing divergence between isolated forms in neighbouring montane forest fragments (e.g. Fjeldså & Lovett 1997; Roy 1997; Fjeldså & Bowie 2008). Additional highland taxa, *Z. senegalensis jacksoni* and *Z. senegalensis stierlingi*, also occupy montane forest habitats throughout Kenya and Tanzania; however, their presence in multiple nonconnected forest fragments indicates that unlike *Z. poliogaster*, highland *Z. senegalensis* populations were not restricted by low dispersal abilities.

Our results provide strong support for mechanisms founded on niche divergence. Both the gradient speciation model and the vanishing refuge model have previously been used to explain the occurrence of sister taxa in adjacent but distinct habitats (e.g. Vanzolini & Williams 1981; Moritz *et al*. 2000; Ogden & Thorpe 2002; Hall 2005; Kozak & Wiens 2007), but in the absence of data on the historical rate of gene flow, it is difficult to distinguish between these two alternative hypotheses (Moritz *et al*. 2000). While relationships within clade A1 seemingly support a gradient speciation model, those of clades A2 and B1 are more complicated. For example, the broad lowland distributions of *Z. pallidus* and *Z. s. senegalensis* are not parapatric with respect to their range-restricted sister taxa, *Z. poliogastrus winifredae* and *Z. p. kikuyensis*, and thus, strong directional selection between habitat types along an altitudinal gradient is not supported (Moritz *et al*. 2000; Kozak & Wiens 2007). Additionally, the presence of highland *Z. senegalensis* forms also conflicts with the main predictions of the gradient speciation model, in that taxa should occur in distinct habitats that have altitudinal nonoverlapping geographical distributions (Moritz *et al*. 2000). These sets of relationships, along with several instances of montane forest taxa ancestral to lowland taxa, instead lend some support to the vanishing refuge model (Vanzolini & Williams 1981; Moritz *et al*. 2000). With their exceptional dispersal abilities and rapid diversification, support for an ecological model of speciation in white-eyes may be a consequence of intrinsic factors as opposed to any common biogeographic pattern. Certainly, it is increasingly apparent from comparative regional studies that no single model or geological period can realistically explain the origin and diversification of the exceptional Afromontane biota.

### Recent colonization of Africa and rapid diversification

The phylogenetic hypothesis (supported by mtDNA and AFLPs) identifies a single colonization event of the African continent by the Zosteropidae that, based on an island calibration, is suggested to have occurred as little as 1.55 Ma (95% HPD: 0.97–2.5 Ma) during the latest Pliocene to Early Pleistocene. The divergence into two principal African clades occurred soon after the arrival of *Zosterops* to the African continent, with subsequent rapid diversification suggested to have occurred in the Lower Pleistocene. Independent lineages diversified into differing habitats across sub-Saharan Africa, as well as colonizing the Gulf of Guinea Islands and making a second colonization of Indian Ocean islands (see Warren *et al*. 2006 and Melo *et al*. 2011).

If correct, this time frame, and also that estimated using the conservative avian molecular clock, indicates that *Zosterops* colonized Africa well after the fragmentation of montane forests during the Early Oligocene due to the onset of aridification (Lovett 1993; Sepulchre *et al*. 2006). Despite the long-term drying trend of the Plio-Pleistocene climate that reduced forest cover, there have been short alternating periods of extreme humidity and aridity (deMenocal 1995; Trauth *et al*. 2007). These warmer and wetter periods occurred from approximately 2.7–2.5, 1.9–1.7, 1.1–0.9 Ma (Trauth *et al*. 2005) and are assumed to have enabled the relic montane forests to expand to lower altitudes, possibly enabling isolated forest patches to have become connected and presenting opportunities for previously allopatric populations to mix; by contrast, cooler and drier periods are postulated to have led to ecological fragmentation with subsequent genetic isolation of montane forest restricted species (deMenocal 1995). Climatic stability of the highland refugia, in conjunction with repeated climate fluctuations affecting lowland areas, may have played an integral role in the diversification of Montane white-eyes, as suggested for other Eastern Afromontane biota (e.g. Roy 1997; deMenocal 2004; Lawson 2010; Measey & Tolley 2011).

Divergence time estimates for our data based on the use of a volcanic island calibration are approximately 4 times younger than those obtained when applying the 2.1% clock rate highlighting the disparity between these two approaches. Our time frame based on an island calibration supports previous estimates of the molecular rate of evolution in Zosteropidae that have documented significantly faster rates of evolution than the avian molecular clock rate (Warren *et al*. 2006; Moyle *et al*. 2009; Melo *et al*. 2011). Support for use of the age of Grande Comore to date divergences within African *Zosterops* comes from previous studies using geological calibrations that demonstrate consistency in divergence estimates when using different taxon sets, genetic markers and independent calibration points (e.g. New Georgia Group, Soloman Island), as well as different analytical methods (Moyle *et al*. 2009; Melo *et al*. 2011). For example, Moyle *et al*. (2009) dated the divergence of the Gulf of Guinea ‘mainland’ (GGM) clade between 0.89 and 1.35 Ma, which is extremely similar to the estimate produced by the island-calibrated approach used in this study (0.51–1.49 Ma).

Previous phylogenetic avian studies of this region have relied on the avian molecular clock (∼2%) to estimate sequence divergence times (e.g. Roy 1997; Roy *et al*. 2001; Fjeldså & Bowie 2008), although only Voelker *et al*.'s (2010) study has applied this rate using quantitative methods (e.g. nonparametric rate smoothing) making comparisons of divergence estimates between studies problematic. Unsurprisingly, by applying the conservative 2.1% clock rate to our data (Fig. S1), we estimate a similar time frame to Voelker *et al*.'s (2010) study on African forest robins who suggest Pliocene forest retraction c. 5–3 Ma as facilitating diversification in Africa's avifauna. The reliance on a molecular clock for the majority of avian studies is unfortunately due to the paucity of suitable fossil or geological calibrations. However, with broader taxonomic mitogenomic studies (e.g. Pacheco *et al*. 2011) identifying great variation of mutation rates within different bird groups, there is increasing support questioning a universal mitochondrial avian molecular clock (e.g. García-Moreno 2004; Lovette 2004). Nevertheless, time frames based on island ages are not without uncertainty (see Heads 2011) as these studies assume that the island endemic evolved in situ. This scenario does not account for endemic taxa being older than the island they inhabit having survived on nearby islands or mainland and later going extinct there (Heads 2011), and thus, our results based on a single island calibration should be viewed cautiously.

### Underestimation of sky island diversity

Moreau's (1957) assessment that 10% of *Zosterops* species-level diversity occurs on continental landmasses would appear an underestimation, as our results reveal significant nonmonophyly of mainland Africa taxa, specifically endemic montane *Z. poliogastrus* and the widespread *Z. senegalensis*. Based on external morphological data, notably plumage, the montane populations of *Z. poliogastrus* have been classified as subspecies of a wider species complex (Dickinson 2003; van Balen 2008). However, the extensive sampling in this study of five of the eight *Z. poliogastrus* subspecies demonstrates this species to be polyphyletic. Strong support based on mitochondrial and AFLP data of the subspecific montane populations in these polyphyletic species indicates that populations should be elevated to species rank as independent taxonomic units rather than remain intraspecific taxa. As well as being scientifically interesting, this finding is important to considerations of species vulnerability, as conservation organizations (e.g. Birdlife International 2014) follow classifications of these polytypic species simply as ‘*Z. poligastrus*’ (currently considered to be a species of least concern) and thus incorrectly assume range size. The conservation status of these species should therefore be reassessed, particularly as the EABH is currently experiencing severe habitat loss (Myers *et al*. 2000).

Mitochondrial data also identify the polyphyly of the widespread African species *Z. senegalensis* and the northern East Africa species *Z. abyssinicus*, although denser sampling of subspecies is needed to determine a more complete picture of intraspecific relationships. Overall, our results question the utility in *Zosterops* of the traditional phenotypical characters used to delineate bird species. This highlights the need for a complete molecular systematic review of African *Zosterops*, applying species delimitation methods to quantitatively infer taxonomic boundaries (*cf* Fujita *et al*. 2013).

### The use of AFLPs for avian phylogenetic studies

Given the lack of phylogenetic resolution of the ncDNA marker TGFß2, AFLPs were selected here as potentially suitable nuclear markers due to their utility in resolving rapidly evolving vertebrate clades (e.g. Sullivan *et al*. 2004; Joyce *et al*. 2011) as previous studies estimated very recent divergence dates for *Zosterops* (e.g. Moyle *et al*. 2009). However, the AFLP data proved largely disappointing for resolving internal relationships within the African radiation compared to the mtDNA data. While AFLPs have been increasingly used to resolve recently diverged fish radiations (e.g. Sullivan *et al*. 2004; Joyce *et al*. 2011), the use of AFLPs in avian phylogenetic studies is scarce (but see Humphries & Winker 2010), albeit they have been used successfully at the population level (e.g. Parchman *et al*. 2006; Mila *et al*. 2010). Although our AFLP matrix may have contained limited signal with only 255 AFLP loci, another study (Dasmahapatra *et al*. 2009) using a similar sized data matrix (310 AFLP loci) generated a well-resolved tree. Inspection of our data reveals that a large proportion of characters corresponded to alleles that were specific to a single population (private alleles). The larger number of private alleles in the data is likely to have resulted in the strong support for the monophyly of independent populations, specifically range-restricted taxa, with limited phylogenetic resolution between populations. As such, our data compared to those of a similar size (e.g. Dasmahapatra *et al*. 2009) are likely to have underperformed due to a much lower information content as opposed to insufficient data. However, a recent study focusing on auklet relationships (Humphries & Winker 2010) using AFLPs was also unable to generate a well-supported tree despite their data containing a greater number of polymorphic sites than did ours. It is possible that both of these avian studies selected taxa that are too divergent or that the stochastic process of incomplete lineage sorting has masked any phylogenetic signal (Humphries & Winker 2010). More avian studies using AFLPs are needed to determine the utility of these markers for phylogenetic inference.

## Conclusion

Understanding how highly fragmented landscapes have influenced population differentiation and speciation is important not only regarding evolutionary processes responsible for generating elevated diversity, but in quantifying the biodiversity present for future conservation planning. This is particularly important in the Eastern Afromontane region that contains several thousand endemic species, but which is threatened by habitat loss and climate change. Our genetic study focusing on endemic Eastern Afromontane white-eyes indicates that contrary to other studies on unrelated taxonomic groups, niche divergence was likely as a major driver of speciation in this very recent clade. However, although our data support a speciation model in which reproductive isolation accumulates in allopatry with a significant contribution from ecologically mediated divergent natural selection, we are unable to test between different ecological models of speciation (i.e. gradient and vanishing speciation hypotheses). In addition to our findings highlighting how a highly fragmented landscape impacts speciation processes, we suggest that under a revised taxonomy, a number of East African sky islands would gain a new endemic *Zosterops* species. Based on our findings, montane *Zosterops* should be re-evaluated regarding their conservation status, particularly given the vulnerability of this biodiversity hotspot. These results further highlight the need for additional phylogeographic studies of taxa from this region to assess its unique diversity.

## Data accessibility

DNA sequences: GenBank accession nos LK056696–LK056918.

Phylogenetic data matrices (mtDNA and AFLP) and bird specimen photographs have been deposited in the Dryad data repository (doi:10.5061/dryad.st56 h).

## Supporting Information

Appendix S1 Details of AFLP amplification and scoring.

Fig S1 Divergence estimates for *Zosterops* generated using BEAST based on the 2.1% avian molecular clock for the Cyt *b* dataset. 95% highest posterior density (HPD) bars in grey, while those in red are superimposed from Fig. 3 (i.e. based on the volcanic island-calibrated timetree).

Table S1 Taxa, sampling locations, altitude and Genbank accession numbers used in this study.

Table S2 Primers and thermal cycling conditions used in amplification and sequencing of genes.

## Abbreviations

J.J.D., S.C.C. and R.P.P-J.: designed the study.
J.J.D. and S.C.C.: wrote the manuscript with input from R.P.P-J.
S.C.C., J.C.H. and B.A.: conducted the fieldwork.
S.C.C.: performed the laboratory work. J.J.D.
S.C.C.: analysed the data.
