# Supplementary material for: Niche divergence promotes rapid diversification of East African sky island white-eyes (Aves: Zosteropidae)
Source: Mol Ecol. 2014 Jul 8;23(16):4103–18. doi: 10.1111/mec.12840 (PMC4255762; doi:10.1111/mec.12840)
Supplement: Appendix S1 — Details of AFLP amplification and scoring. [file mec0023-4103-SD1.pdf]

Appendix S1 Details of AFLP amplification and scoring.

Amplification

Pre-selective amplifications were performed with 4µl of ligation product, 6.72µl ddH2O, 2µl 10X PCR buffer (Bioline), 1µl MgCl2 (25µM), 2µl dNTPs (2.5 µM of each), 1µl BSA (10 mg/ml), 0.6µl of each pre-selective primer (10µM), and 0.08µl of *Taq* DNA polymerase (0.4 units). PCR cycling parameters were a preliminary 72℃ extension for 2 minutes followed by 20 cycles of 20 seconds at 94℃, 30 seconds at 56℃ and 2 minutes at 72℃, with a final hold at 60℃ for 30 minutes. Following pre-amplification PCR products were diluted 1:5 with ddH2O. Selective amplification reactions were performed in 10µl reaction volumes adding 5µl of the diluted (1:5) pre-selective PCR product to 1.04µl ddH2O, 2.0µl dNTPs (2.5µM of each), 1µl 10 X PCR buffer (Bioline), 0.3µl MgCl2 (25µM), 0.06µl *Taq* DNA polymerase (0.3 units) and 0.3µl of both MseI and EcoRI selective amplification primers (10µM). Thermal cycling conditions for selective PCR consisted of 2 min at 94°C followed by 10 cycles with 20 sec at 94°C, 30 sec at annealing temperature, which decreased in each cycle by 1°C from 65°C to 56°C, and 2 min at 72°C. The PCR continued for 25 cycles with 20 sec at 94°C, 30 sec at 56°C and 2 min at 72°C, followed by a holding step at 60°C for 30 min. Five individuals were repeated from the restriction ligation stage onwards to obtain a relative assessment of the repeatability of AFLP profiles.

To account for differential amplitude of emissions between dyes (fluorophores), five samples were run using a series of dilution ratios (neat, 1:5 1:10) for each primer combination. From these dye ratio tests, an RFU emission standard could be identified for each dye, from which the optimum-pooling ratio could be calibrated.

AFLP scoring

The AFLP samples were then re-analysed using the preliminary analysis parameters to remove much of the poor bin assignment before manual inspection. The resulting AFLP profiles were over-laid and the quality, position and width of each bin relative to the concatenated profile were manually assessed. For each fragment, bins were saved, modified or deleted depending on their fit to the data, with only unambiguously scorable loci (bins) retained for future analysis. In addition, bins corresponding to fragments less that 75bp were removed and peaks found in less than 2% of samples were discounted.

AFLP reads were subsequently re-analysed using the manually edited panel and an amplitude threshold that was approximately half that of the original estimated value. Given the variation in peak height both across samples and between bins, each bin within the panel was re-assessed and all fragments within each bin were manually scored using an independent bin-specific amplitude threshold. This threshold was identified by overlaying all peaks within each bin and assessing the signal to noise ratio.
