# Supplementary material for: Niche divergence promotes rapid diversification of East African sky island white-eyes (Aves: Zosteropidae)
Source: Mol Ecol. 2014 Jul 8;23(16):4103–18. doi: 10.1111/mec.12840 (PMC4255762; doi:10.1111/mec.12840)
Supplement: Fig S1 — Divergence estimates for Zosterops generated using BEAST based on the 2.1% avian molecular clock for the Cyt b dataset. 95% highest posterior density (HPD) bars in grey, while those in red are superimposed from Fig. 3 (i.e. based on the volcanic island-calibrated timetree). [file mec0023-4103-SD2.pdf]

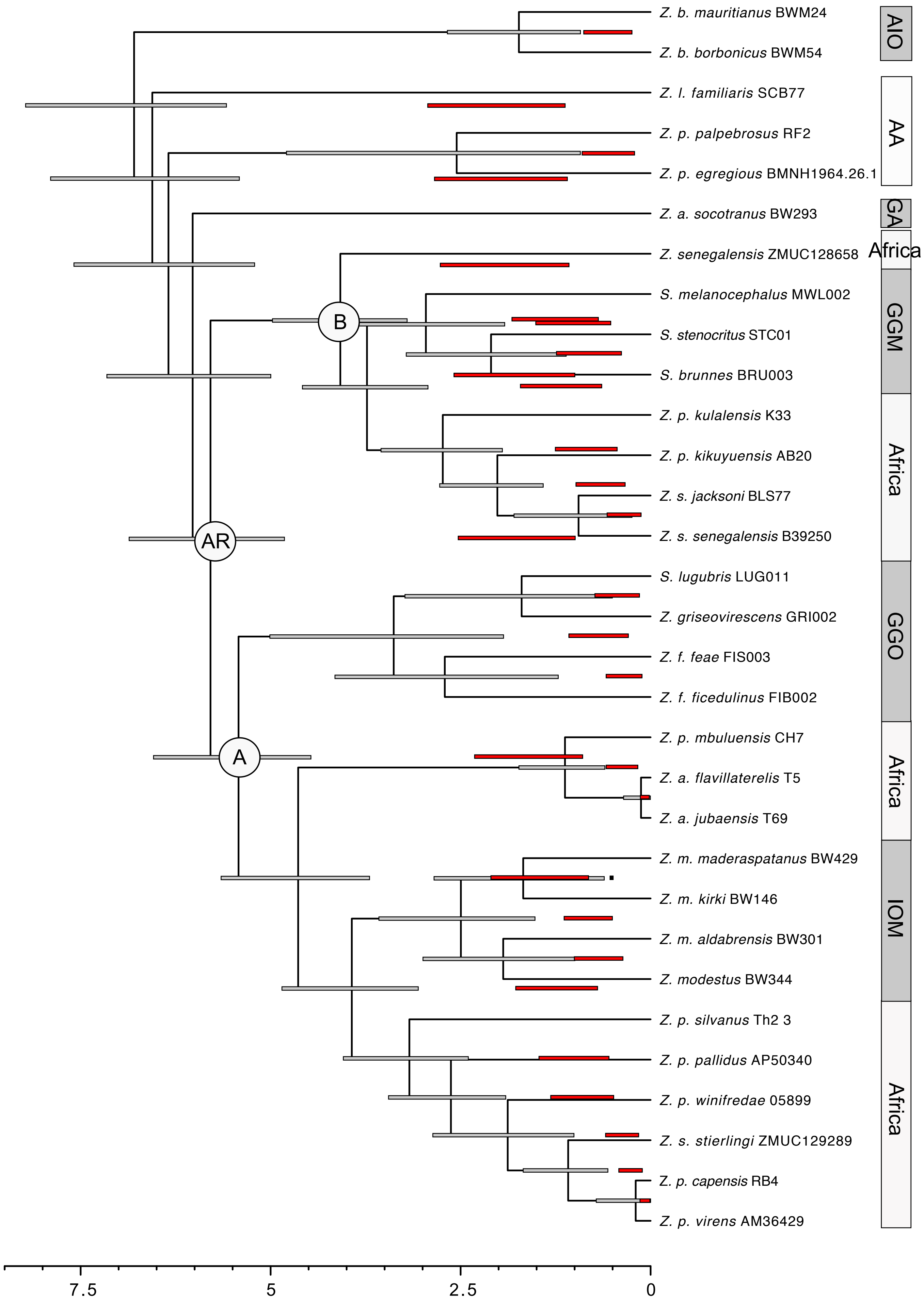

Fig. S1 Divergence estimates for *Zosterops* generated using BEAST based on the 2.1% avian molecular clock for the Cyt *b* dataset. 95% Highest Posterior Density (HPD) bars in grey, while those in red are superimposed from Fig. 3 (i.e. based on the volcanic island calibrated timetree).
