# Supplementary material for: Niche divergence promotes rapid diversification of East African sky island white-eyes (Aves: Zosteropidae)
Source: Mol Ecol. 2014 Jul 8;23(16):4103–18. doi: 10.1111/mec.12840 (PMC4255762; doi:10.1111/mec.12840)
Supplement: Table S1 — Taxa, sampling locations, altitude and Genbank accession numbers used in this study. [file mec0023-4103-SD3.pdf]

Table S1 Taxa, sampling locations, altitude and Genbank accession numbers used in this study.

Taxon	Locality	Altitude (m)	Sample code	Source	Photo	AFLP	ND3	Cyt *b*
Zosterornis ***whiteheadi***	Phillippines		KUNHM 18001	NCBI			JN826960	JN8272210
Zosterops								
Z. palpebrosus								
palpebrosus	Nepal		RF2	NCBI			DQ328448	DQ328348
egregious	Sasan, Kathiawar, Gujarat, India		BMNH 1964.26.1	NCBI			DQ328450	DQ328350
Z. lateralis	Brisbane, Australia		SCB77	NCBI			DQ328488	DQ328388
Z. abyssinicus								
socotranus	Socotra, Gulf of Aden		BW292	WARREN		✓	LK056917	LK056803
socotranus	Socotra, Gulf of Aden		BW293	WARREN		✓	LK056918	LK056804
jubaensis	South Horr, Kenya	1065	T60	COX	✓	✓	LK056844	LK056734
jubaensis	South Horr, Kenya	1065	T61	COX	✓	✓	LK056847	LK056737
jubaensis	South Horr, Kenya	1065	T65	COX	✓	✓	LK056848	LK056738
jubaensis	South Horr, Kenya	1065	T69	COX	✓	✓	LK056845	LK056735
jubaensis	South Horr, Kenya	1065	T70	COX	✓	✓	LK056849	LK056739
jubaensis	South Horr, Kenya	1065	T73	COX	✓	✓	LK056846	LK056736
jubaensis	South Horr, Kenya	1065	T76	COX	✓		LK056840	
jubaensis	South Horr, Kenya	1065	T77	COX	✓	✓	LK056841	LK056733
jubaensis	South Horr, Kenya	1065	T84	COX	✓		LK056843	
jubaensis	South Horr, Kenya	1065	T85	COX	✓		LK056842	
flavilateralis	Umani spring, Kenya	927	T4	COX	✓		LK056831	LK056724
flavilateralis	Umani spring, Kenya	927	T5	COX	✓	✓	LK056830	LK056723
flavilateralis	Umani spring, Kenya	927	T11	COX	✓	✓	LK056832	LK056725
flavilateralis	Umani spring, Kenya	927	T14	COX	✓		LK056836	LK056729
flavilateralis	Umani spring, Kenya	927	T15	COX	✓	✓	LK056837	LK056730
flavilateralis	Umani spring, Kenya	927	T17	COX	✓	✓	LK056833	LK056726

flavilateralis	Umani spring, Kenya	927	T20	COX	✓	✓	LK056834	LK056727
flavilateralis	Umani spring, Kenya	927	T21	COX	✓	✓	LK056838	LK056731
flavilateralis	Umani spring, Kenya	927	T23	COX	✓		LK056835	LK056728
flavilateralis	Umani spring, Kenya	927	T30	COX	✓	✓	LK056839	LK056732
flavilateralis	Nairobi, Kenya		RB3	NCBI			DQ328439	DQ328339
Z. pallidus								
pallidus	South Africa		AP50340	PFIAO		✓	LK056910	LK056796
capensis	Cape Town, South Africa		RB4	PFIAO			LK056905	LK056791
capensis	Cape Town, South Africa		RB1	PFIAO			LK056906	LK056792
capensis	Cape Town, South Africa		RB4	PFIAO			LK056905	LK056791
capensis	Cape Town, South Africa		K1	PFIAO		✓		
virens	South Africa		AM36429	PFIAO		✓	LK056907	LK056793
virens	South Africa		AM36433	PFIAO		✓	LK056908	LK056794
Z. senegalensis								
senegalensis	Northern Region, Ghana		B39250	NCBI			DQ328443	DQ328343
senegalensis	Northern Region, Ghana		B39514	NCBI			DQ328442	DQ328342
senegalensis	Rwenzori, Uganda		ZMUC145103	ZMUC		✓		
senegalensis	Rwenzori, Uganda		ZMUC145211	ZMUC		✓		
stenocricotus	Mt Cameroon, Cameroon		STC01	MELO		✓	LK056895	LK056782
stenocricotus	Mt Cameroon, Cameroon		STC03	MELO		✓	LK056896	
jacksoni	Mathews Range, Kenya	1600	BLS06	BORGHESIO		✓	LK056873	LK056762
jacksoni	Mt Nyiru, Kenya	2600	BLS35	BORGHESIO			LK056883	LK056772
jacksoni	Mt Nyiru, Kenya	2600	BLS36	BORGHESIO		✓		
jacksoni	Mt Nyiru, Kenya	2600	BLS65	BORGHESIO		✓	LK056882	LK056771
jacksoni	Mathews Range, Kenya	1600	BLS77	BORGHESIO		✓	LK056871	LK056760
jacksoni	Mathews Range, Kenya	1600	BLS81	BORGHESIO		✓	LK056872	LK056761
jacksoni	Mt Nyiru, Kenya		ZMUC 131316	ZMUC		✓	LK056889	

jacksoni	Mt Nyiru, Kenya		ZMUC 131317	ZMUC		✓	LK056886	LK056774
jacksoni	Mt Nyiru, Kenya		ZMUC 131324	ZMUC			LK056885	LK056773
jacksoni	Mt Nyiru, Kenya		ZMUC 131325	ZMUC			LK056884	
jacksoni	Mt Nyiru, Kenya		ZMUC 131331	ZMUC		✓	LK056887	LK056779
jacksoni	N. Aberdares, Kenya		ZMUC 146780	ZMUC			LK056888	LK056775
jacksoni	N. Aberdares, Kenya		ZMUC 146784	ZMUC			LK056890	LK056777
jacksoni	N. Aberdares, Kenya		ZMUC 146785	ZMUC		✓	LK056891	LK056778
jacksoni	N. Aberdares, Kenya		ZMUC 146786	ZMUC		✓		LK056776
jacksoni	N. Aberdares, Kenya		ZMUC 146787	ZMUC		✓		
jacksoni	Kakamega, Kenya	1627	T41	COX	✓	✓	LK056874	LK056763
jacksoni	Kakamega, Kenya	1627	T42	COX	✓	✓	LK056878	LK056767
jacksoni	Kakamega, Kenya	1627	T49	COX	✓	✓	LK056875	LK056764
jacksoni	Kakamega, Kenya	1627	T50	COX	✓		LK056877	LK056766
jacksoni	Kakamega, Kenya	1627	T51	COX	✓		LK056881	LK056770
jacksoni	Kakamega, Kenya	1627	T52	COX	✓		LK056879	LK056768
jacksoni	Kakamega, Kenya	1627	T53	COX	✓	✓	LK056880	LK056769
jacksoni	Kakamega, Kenya	1627	T54	COX			LK056876	LK056765
stierlingi	Tabora, Tanzania		ZMUC 145467	ZMUC		✓	LK056897	LK056783
stierlingi	Udzungwa Mts, Tanzania		ZMUC 140192	ZMUC		✓	LK056901	LK056787
stierlingi	Poroto Mts, Tanzania		ZMUC 142605	ZMUC			LK056902	LK056788
stierlingi	Poroto Mts, Tanzania		ZMUC 142607	ZMUC		✓	LK056898	LK056784
stierlingi	W. Usambara Mts, Tanzania		ZMUC 129298	ZMUC		✓	LK056900	LK056786
stierlingi	W. Usambara Mts, Tanzania		ZMUC 129289	ZMUC			LK056899	LK056785
stierlingi	Simba Forest, West Kilimanjaro, Tanzania		ZMUC 121003	ZMUC		✓		
stierlingi	Simba Forest, West Kilimanjaro , Tanzania		ZMUC 121004	ZMUC		✓		
stierlingi	Uhafiwa, Tanzania		O8255	NCBI			DQ328438	DQ328338
sp.	Kivu, Democratic Republic of the Congo		ZMUC 128660	ZMUC			LK056893	LK056780
sp.	Kivu, Democratic Republic of the		ZMUC 128632	ZMUC			LK056892	LK056779

	Congo							
sp.	Kivu, Democratic Republic of the Congo		ZMUC 128658	ZMUC			LK056894	LK056781
Z. poliogastrus								
kulalensis	Mt Kulal, Kenya	1932	K30	COX	✓	✓	LK056819	LK056711
kulalensis	Mt Kulal, Kenya	1932	K31	COX	✓		LK056822	LK056714
kulalensis	Mt Kulal, Kenya	1932	K33	COX	✓	✓	LK056816	LK056708
kulalensis	Mt Kulal, Kenya	1932	K34	COX	✓	✓	LK056821	LK056713
kulalensis	Mt Kulal, Kenya	1932	K35	COX	✓	✓	LK056817	LK056709
kulalensis	Mt Kulal, Kenya	1932	K37	COX	✓	✓	LK056818	LK056710
kulalensis	Mt Kulal, Kenya	1932	K38	COX	✓	✓	LK056823	LK056715
kulalensis	Mt Kulal, Kenya	1932	K39	COX	✓	✓	LK056824	LK056716
kulalensis	Mt Kulal, Kenya	1932	K41	COX	✓		LK056820	LK056712
kulalensis	Mt Kulal, Kenya	1932	K42	COX	✓	✓	LK056825	LK056717
kulalensis	Mt Kulal, Kenya	1800	2MK3	HABEL		✓	LK056826	LK056718
kulalensis	Mt Kulal, Kenya	1800	2MK7	HABEL			LK056827	LK056719
kulalensis	Mt Kulal, Kenya	1800	2MK8	HABEL			LK056828	LK056720
kulalensis	Mt Kulal, Kenya	1800	2MK9	HABEL			LK056829	LK056721
kulalensis	Mt Kulal, Kenya	1800	2MK10	HABEL				LK056722
kikuyuensis	Mt Kenya, Kenya	2900	MK1	HABEL			LK056859	LK056750
kikuyuensis	Mt Kenya, Kenya	2900	MK2	HABEL			LK056862	LK056752
kikuyuensis	Mt Kenya, Kenya	2900	MK3	HABEL		✓	LK056861	LK056751
kikuyuensis	Mt Kenya, Kenya	2900	MK6	HABEL		✓	LK056863	LK056753
kikuyuensis	Mt Kenya, Kenya	2900	MK7	HABEL			LK056860	
kikuyuensis	Aberdares Range, Kenya	2700	AB2	HABEL		✓	LK056865	
kikuyuensis	Aberdares Range, Kenya	2700	AB4	HABEL		✓	LK056868	LK056757
kikuyuensis	Aberdares Range, Kenya	2700	AB10	HABEL		✓	LK056867	LK056756
kikuyuensis	Aberdares Range, Kenya	2700	AB11	HABEL			LK056869	LK056758
kikuyuensis	Aberdares Range, Kenya	2700	AB12	HABEL		✓	LK056870	LK056759

kikuyuensis	Aberdares Range, Kenya	2700	AB13	HABEL		LK056864	LK056754
kikuyuensis	Aberdares Range, Kenya	2700	AB20	HABEL	✓	LK056866	LK056755
kikuyuensis	Mt Kenya, Kenya		RB2	NCBI		DQ328447	DQ328247
silvanus	Mbololo, Taita Hills, Kenya		08580	NCBI		DQ328437	DQ328337
silvanus	Taita Hills, Kenya	1900	TH 1-2	HABEL		LK056813	LK056704
silvanus	Taita Hills, Kenya	1900	TH 1-3	HABEL	✓	LK056812	LK056703
silvanus	Taita Hills, Kenya	1900	TH 1-10	HABEL	✓	LK056811	LK056702
silvanus	Taita Hills, Kenya	1900	TH 1-13	HABEL	✓		
silvanus	Taita Hills, Kenya	1900	TH 1-21	HABEL	✓		LK056707
silvanus	Taita Hills, Kenya	1900	TH 2-3	HABEL	✓	LK056805	LK056696
silvanus	Taita Hills, Kenya	1900	TH 2-10	HABEL	✓	LK056806	LK056697
silvanus	Taita Hills, Kenya	1900	TH 2-12	HABEL	✓	LK056809	LK056700
silvanus	Taita Hills, Kenya	1900	TH 2-15	HABEL	✓	LK056808	LK056699
silvanus	Taita Hills, Kenya	1900	TH 3-12	HABEL	✓	LK056810	LK056701
silvanus	Taita Hills, Kenya	1900	TH 3-13	HABEL		LK056814	LK056705
silvanus	Taita Hills, Kenya	1900	TH 3-20	HABEL	✓	LK056807	LK056698
silvanus	Taita Hills, Kenya	1900	TH 3-21	HABEL	✓	LK056815	LK056706
mbuluensis	Chyulu Hills, Kenya	1900	CH1	HABEL	✓	LK056852	LK056742
mbuluensis	Chyulu Hills, Kenya	1900	CH3	HABEL		LK056854	LK056744
mbuluensis	Chyulu Hills, Kenya	1900	CH6	HABEL	✓	LK056853	LK056743
mbuluensis	Chyulu Hills, Kenya	1900	CH7	HABEL	✓	LK056850	LK056740
mbuluensis	Chyulu Hills, Kenya	1900	CH8	HABEL	✓	LK056851	LK056741
mbuluensis	Chyulu Hills, Kenya	1900	2CH9	HABEL	✓	LK056855	LK056745
mbuluensis	Chyulu Hills, Kenya	1900	2CH11	HABEL	✓	LK056856	LK056746
mbuluensis	Chyulu Hills, Kenya	1900	2CH10	HABEL	✓		LK056747
mbuluensis	Chyulu Hills, Kenya	1900	2CH12	HABEL	✓	LK056857	LK056748
mbuluensis	Chyulu Hills, Kenya	1900	2CH13	HABEL	✓	LK056858	LK056749

winifredae	South Pare Mts, Tanzania	O5899	NCBI		DQ328436	DQ328336
Z. borbonicus						
borbonicus	La Réunion, Mascarenes	BWM46	NCBI	✓	DQ328497	DQ328397
borbonicus	La Réunion, Mascarenes	BWM47	NCBI		DQ328496	DQ328396
borbonicus	La Réunion, Mascarenes	BWM54	WARREN	✓	LK056916	LK056802
mauritianus	Mauritus, Mascarenes	BWM17	WARREN	✓	LK056914	LK056800
mauritianus	Mauritus, Mascarenes	BWM25	WARREN	✓	LK056915	LK056801
mauritianus	Mauritus, Mascarenes	BWM24	WARREN		LK056913	LK056799
Z. ficedulinus						
ficedulinus	Annobón, Gulf of Guinea	FIP002	NCBI		GU827278	GU827230
feae	São Tomé, Gulf of Guinea	FIS003	NCBI		GU827280	GU827232
Z. griseovirescens	Príncipe Island, Gulf of Guinea	GRI002	NCBI		GU827282	GU827234
Z. maderaspatanus						
aldabrensis	Aldabra, Aldabra Group, Seychelles	BW301	NCBI		DQ328487	DQ328387
maderaspatanus	Mt Ankaratra, Madagascar	BW429	WARREN		LK056911	LK056797
maderaspatanus	Mt Ankaratra, Madagascar	BW445	WARREN		LK056912	LK056798
kirki	Grande Comore, Comoros	BW146	NCBI		DQ328476	DQ328376
Z. modestus	Conception, Seychelles	BW344	NCBI		DQ328463	DQ328363
Speirops						
S. brunneus	Bioko, Gulf of Guinea	BRU003	NCBI		GU827268	GU827220
S. melanocephalus	Mt Cameroon, Cameroon	MEL002	NCBI		GU827266	GU827218
S. lugubris	São Tomé, Gulf of Guinea	LUG001	MELO		LK056904	LK056790
S. lugubris	São Tomé, Gulf of Guinea	LUG011	MELO	✓	LK056903	LK056789

Institutional abbreviations
BMNH, Natural History Museum, London; KUNHM, University of Kansas Natural History; PFIAO, Percy FitzPatrick Institute of African Ornithology, South Africa; ZMUC, Zoological Museum, University of Copenhagen.

Sampling
Within East Africa, 51 *Z. poliogastrus* samples were collected from five isolated montane forests, giving an excellent coverage of the restricted distributions of the four subspecies: *Z. p. silvanus* (Taita Hills and Mt Kasigau), *Z. p. mbuluensis* (Chyulu Hills and Ol Doinyo Orok), *Z. p. kulalensis* (Mt Kulalensis) and *Z. p. kikuyensis* (Central Kenyan Highlands) (Fig. 1C). Additional sequences for *Z. p. winifredae* (1 individual) were obtained from Genbank, enabling the phylogenetic assessment of five of the eight recognised *Z. poliogaster* subspecies (Dickinson 2003). Unfortunately it was not possible to obtain samples for three *Z. poliogastrus* subspecies represented by the two Ethiopian subspecies (*Z. p. poliogaster* and *Z. p. kaffensis*) and one Tanzanian subspecies (*Z. p. eurycricotus*). A further 29 samples of *Z. senegalensis* from higher elevations populations were sampled, including four populations of *Z. s. jacksoni* (Kenya) and three populations of *Z. s. stierlingi* (Tanzania). Within Kenya an additional 21 samples of *Z. abyssinicus* (White-breasted white-eye) were collected from lowland scrub and riverine areas. These represented two (*Z. a. flavilateralis* and *Z. a. jubaensis*) of the four described mainland African *Z. abyssinicus* subspecies that are found throughout the lowlands of North East Africa.

Taxa from outside the focal region include represent an insular *Z. abyssinicus* race from the Island of Socotra, Gulf of Aden (*Z. a. socotranus*), three subspecies of *Z. pallidus* from South Africa (*Z. p. pallidus*, *Z. p. capensis*, *Z. p. virens*), *Z. senegalensis* from the Democratic Republic of Congo (not identified to the sub-specific level) and two *Z. senegalensis* subspecies from Ghana, Cameroon and Uganda (*Z. s. senegalensis*, *Z. s. stenocritus*). In addition to continental African taxa, representatives from Atlantic (Gulf of Guinea) and Indian Ocean islands systems are included based on the results of previous studies (Warren *et al*. 2006; Melo *et al*. 2011), as well as Asian (*Z. palpebrosus*) and Australian (*Z. lateralis*) species. The Chestnut-faced babbler, *Zosterornis whiteheadi* (Zosteropidae) (previously diagnosed as *Stachyris whiteheadi*) was selected as an outgroup based on previous studies (e.g. Warren *et al.* 2006; Melo *et al.* 2011).
