# Supplementary material for: Niche divergence promotes rapid diversification of East African sky island white-eyes (Aves: Zosteropidae)
Source: Mol Ecol. 2014 Jul 8;23(16):4103–18. doi: 10.1111/mec.12840 (PMC4255762; doi:10.1111/mec.12840)
Supplement: Table S2 — Primers and thermal cycling conditions used in amplification and sequencing of genes. [file mec0023-4103-SD4.pdf]

Table S2 Primers and thermal cycling conditions used in amplification and sequencing of genes.

Gene	Primer name	Primer sequence	Reference	PCR conditions			
				Denature	Annealing	Extension	Cycles
ND3	L10755<br>H11151	5’-GACTTCCAATCTTTAAAATCTG-3’<br>5’-GATTTGTTGAGCCGAAATCAAC-3’	Chesser (1999)	94°C 30s	58°C 40s	72°C 40s	35
Cyt-b	CytbF25	5’ GGCTCTCAATCTTCGTAAAAACC-3’	This study	94°C 30s	60°C 60s	72°C 90s	35
	CytbR649	5’-GGGTGGAATGGGATTTTGTC-3’	This study				
	CytbF409	5’-GTAGGCTACGTCCTACCCTGAG-3’	This study				
	CytbH16065	5’-GAGTCTTCAGTCTCTGGTTTACAAGAC-3	Helm-Bychowski & Cracraft (1993)				
TFGß2	TGF5	5’-GAAGCGTGCTCTAGATGCTG-3’	Primmer *et al.* (2002)	94°C 30s	56°C 30s	72°C 45s	35
	TGF6	5’-AGGCAGCAATTATCCTGCAC-3’					

PCR amplifications were performed in 15μl volumes with 2μl total genomic DNA, 9.7μl ddH_2O, 1.5μl 10X PCR buffer, 0.75μl MgCl2 (50mM), 0.15μl dNTP (2.5 mM of each), 0.45μl of each primer (10mM), and 0.15μl Taq DNA polymerase (5 units/μl).

References

Chesser RT (1999) Molecular systematics of the Rhinocryptid genus *Pteroptochos*. *The Condor*, **101**, 439-446.

Helm-Bychowski K, Cracraft J (1993) Recovering phylogenetic signal from DNA sequences: relationships within the Corvine assemblage (Class Aves) as inferred from complete sequences of the mitochondrial DNA cytochrome-*b* gene. *Molecular Biology and Evolution*, **10**, 1196-1214.

Primmer CR, Borge T, Lindell J, Setre GP (2002) Single-nucleotide polymorphism characterization in species with limited available sequence information: high nucleotide diversity revealed in the avian genome. *Molecular Ecology*, **11**, 603-612.
